# Needs of family caregivers of older adults with post-stroke sequelae

**DOI:** 10.15649/cuidarte.4982

**Published:** 2026-03-26

**Authors:** Gerardo Saucedo-Pahua, María Mercedes Moreno González, María De Jesús Jiménez González, Tirso Duran-Badillo, Clara Teresita Morales Álvarez, Jack Roberto Silva Fhon

**Affiliations:** 1 Universidad de Guanajuato, Instituto Mexicano del Seguro Social, Morelia, México. E-mail: g.saucedopahua@ugto.mx Universidad de Guanajuato Morelia Mexico g.saucedopahua@ugto.mx; 2 Universidad de Guanajuato, Celaya, México. E-mail: ma.moreno@ugto.mx Universidad de Guanajuato Celaya Mexico ma.moreno@ugto.mx; 3 Universidad de Guanajuato, Celaya, México. E-mail: mj.jimenez@ugto.mx Universidad de Guanajuato Celaya Mexico mj.jimenez@ugto.mx; 4 Universidad Autónoma de Tamaulipas, Matamoros, México. E-mail: tduran@docentes.uat.edu.mx Universidad Autónoma de Tamaulipas Matamoros Mexico tduran@docentes.uat.edu.mx; 5 Universidad de Guanajuato, Instituto Mexicano del Seguro Social, Celaya, México. E-mail: tmorales@ugto.mx Universidad de Guanajuato Celaya Mexico tmorales@ugto.mx; 6 Universidade de São Paulo, São Paulo, SP, Brasil. E-mail: betofhon@usp.br Universidade de São Paulo São Paulo Brasil betofhon@usp.br

**Keywords:** Needs Assessment, Caregivers, Hospital to Home Transition, Stroke, Aged, Qualitative Research, Evaluación de Necesidades, Cuidadores, Transición del Hospital al Hogar, Accidente Cerebrovascular, Anciano, Investigación Cualitativa, Avaliação das Necessidades, Cuidadores, Transição do Hospital para o Domicílio, Acidente Vascular Cerebral, Idoso, Pesquisa Qualitativa

## Abstract

**Introduction:**

Stroke is the leading cause of disability, resulting in mobility deficits, cognitive and functional impairments that lead to dependence. As a result, caregivers experience stress, anxiety, and uncertainty during the transition from hospital to home due to inadequate preparation for taking on the role.

**Objective:**

To describe the needs of family caregivers of older adults with post-stroke sequelae.

**Materials and Methods:**

A qualitative, phenomenological study was conducted using in-depth, open-ended interviews, audio-recorded and transcribed, accompanied by field notes. A convenience sample of six participants was selected. An interpretative phenomenological analysis was conducted in light of Edmund Husserl's theoretical and referential framework.

**Results:**

Six participants, daughters of older adults with post-stroke sequelae, took part in the study. Most were married, had a secondary education, were homemakers, and reported low economic income. Three core themes of meaning emerged from their caregiving experiences: Awareness of reality, perceived needs, and caregiving coping strategies. They expressed their feelings and interpretations in detail, sharing from their lived experiences the challenges and care required in response to their family member’s health deterioration.

**Discussion:**

Caregivers face emotional and knowledge-related challenges when caring for older adults after a stroke. According to various studies, professional training and spiritual and family support are key strategies for reducing caregiver burden and improving the quality of care.

**Conclusion:**

The identified needs include caregiving support, highlighting preparation for home care, and access to psychological assistance.

## Resumen

**Introducción:**

El accidente cerebrovascular es la principal causa de discapacidad que genera déficit en la movilidad, alteraciones cognitivas y funcionales que generan dependencia. Como resultado los cuidadores experimentan estrés, ansiedad e incertidumbre durante la transición del hospital al hogar, derivado de la baja preparación para la asunción del rol.

**Objetivo:**

Describir las necesidades de los cuidadores familiares de adultos mayores que presentan secuelas post accidente cerebrovascular.

**Materiales y métodos:**

Estudio cualitativo, fenomenológico, mediante entrevistas abiertas a profundidad grabadas en audio y transcritas, con notas en diario de campo. Muestra de seis personas seleccionadas a conveniencia. Análisis fenomenológico interpretativo a la luz del marco teórico y referencial Edmund Husserl.

**Resultados:**

Participaron seis, hijas de adultos mayores con secuelas de accidente cerebrovascular, la mayoría eran casadas, con educación secundaria, amas de casa y con bajos ingresos económicos. De sus experiencias de cuidado emergieron tres núcleos de sentido: conciencia de la realidad, necesidades y estrategias de afrontamiento para el cuidado. En ellas, expresan a detalle sus sentimientos e interpretaciones, compartiendo desde sus vivencias los desafíos y cuidados que implica el deterioro de salud de un familiar.

**Discusión:**

Los cuidadores enfrentan desafíos emocionales y de conocimiento en el cuidado del adulto mayor con accidente cerebrovascular. La capacitación profesional, el apoyo espiritual y familiar son estrategias clave para reducir la sobrecarga y mejorar la calidad del cuidado según diversos estudios.

**Conclusiones:**

Las necesidades identificadas incluyen el apoyo en el cuidado, destacando la preparación para brindar atención en el hogar y el acceso a ayuda psicológica.

## Resumo

**Introdução:**

O acidente vascular cerebral é a principal causa de incapacidade, levando a déficits de mobilidade e comprometimentos cognitivos e funcionais que geram dependência. Como resultado, os cuidadores vivenciam estresse, ansiedade e incerteza durante a transição do hospital para o lar, decorrentes da preparação inadequada para assumir o papel.

**Objetivo:**

descrever as necessidades de cuidadores familiares de idosos com sequelas pós-acidente vascular cerebral.

**Materiais e métodos:**

Estudo qualitativo fenomenológico, utilizando entrevistas abertas e em profundidade, gravadas em áudio e transcritas, com anotações em diário de campo. Uma amostra de seis pessoas foi selecionada por conveniência. Foi realizada análise fenomenológica interpretativa à luz do referencial teórico de Edmund Husserl.

**Resultados:**

Participaram seis pessoas, filhas de idosos com sequelas pós-acidente vascular cerebral. A maioria era casada, com ensino médio completo, dona de casa e de famílias de baixa renda. Três significados centrais emergiram de suas experiências de cuidado: consciência da realidade, necessidades e estratégias de enfrentamento para o cuidado. Nestes eles expressam seus sentimentos e interpretações em detalhes, compartilhando suas experiências sobre os desafios e o cuidado que acompanham a deterioração da saúde de um membro da família.

**Discussão:**

Os cuidadores enfrentam desafios emocionais e de conhecimento no cuidado de idosos com acidente vascular cerebral. Treinamento profissional, apoio espiritual e familiar e acesso a apoio psicológico são estratégias essenciais para reduzir a sobrecarga e melhorar a qualidade do cuidado, de acordo com diversos estudos.

**Conclusões:**

as necessidades identificadas incluem apoio na prestação de cuidados, destacando a preparação para o atendimento domiciliar e o acesso a ajuda psicológica.

## Introduction

Worldwide, stroke is one of the leading causes of death and long-term disability. The risk of developing this condition increases among people living with hypertension, hyperglycemia, hyperlipidemia, overweight, and obesity^1^. Stroke is characterized by a reduction or interruption of blood supply to a part of the brain. This condition usually occurs when a thrombus forms and obstructs a cerebral blood vessel, depriving that specific region of oxygen and nutrients^2^. As a result, cell death occurs in the affected area, leading to movement deficits (hemiplegia) and cognitive and functional impairments; the severity of these sequelae varies depending on the extent and location of the brain damage^3^.

Worldwide, there are approximately 100 million stroke survivors, more than one-third of whom live with some degree of disability and dependence^4^,^5^, requiring assistance from a family caregiver (FC) who provides home-based care to help them carry out activities of daily living^6^; This work involves providing care and physical and emotional assistance, making decisions that benefit the patient, and taking responsibility for their own self-care^7^.

When assuming the role of FC, needs emerge from perceptions and experiences during caregiving in hospital or home settings; the deficiencies or requirements for performing the role vary according to individual, family, social, and political contexto^8^, “Felt need” is defined as the set of issues and/ or actions experienced by a person who provides non-professional care to a dependent family member at home. These needs arise when support from the healthcare or community system is not received to address the problem^9^. Identifying caregivers' needs will inform the services they desire or require to support their caregiving role from their own perspective. Two systematic reviews of qualitative and quantitative studies provide evidence on caregivers’ needs at specific moments along the caregiving trajectory. Among the main difficulties are the lack of knowledge and skills for home care and rehabilitation, physical and mental self-care, access to adequate services, self-management of emotional and psychological needs, and satisfaction of needs related to relationships and social interaction^10^,^11^, as well as the lack of family, friend, and community support to supplement this role^12^.

In 2020, da Silva et al.^4^ described the needs of FCs in their context as guidance, care modeling, and self-care strategies. Tyagi et al.^13^ in 2021, pointed out the importance of caregiver self-management, identifying different faith-based coping and distancing methods, but also reporting emotional stress. In this respect, Tamayo et al.^14^ in 2022 highlighted the importance of developing skills that improve effective communication between the caregiver and the patient, as well as making architectural modifications to the home environment to increase patient comfort and safety. Additionally, Heim et al.^15^ in 2023 described the importance of addressing negative feelings, such as fear, anxiety, and loneliness, which could emerge during the transition to the caregiver role.

The needs of caregivers of older adults with post-stroke sequelae are diverse, complex, and difficult to anticipate and fulfill. This diversity is influenced by each country’s specific context. In Mexico, to date, no research has been identified that explores the needs of caregivers at any stage of stroke recovery.

The results presented here contribute significantly to strengthening nursing care by identifying caregiver needs from a comprehensive approach that encompasses the patient and their family environment. This understanding will make it possible to design caregiver training programs, promote coping strategies, improve the transition from hospital to home care, and prevent caregiver burden.

The objective of the study was to describe the needs of family caregivers of older adults with post- stroke sequelae.

## Materials and Methods

The study is grounded in the interpretive paradigm^16^ and used a qualitative phenomenological design^17^ informed by Edmund Husserl’s philosophical framework^18^, as it seeks to describe and understand the individually lived experience from the participant's reflective capacity in relation to the phenomenon under study^19^.

To obtain the information, in-depth, open-ended interviews were used as the qualitative data collection technique. These interviews were conducted in the Internal Medicine Service of the Hospital General Regional Número Uno of the Mexican Social Security Institute (IMSS, by its acronym in Spanish) in Michoacán, Mexico, between July and September 2024.

Six FCs of older adults living with physical or cognitive post-stroke sequelae were included, selected through convenience sampling^20^. FCs included were over 18 years of age, had at least an elementary school education, provided unpaid care, with caregiving shifts exceeding five hours at least twice a week, and had performed the caregiving role for more than six months. Two participants who initially met the inclusion criteria and were interviewed were excluded from the study because the relatives they were caring for passed away, preventing subsequent review and verification of the collected data.

Each interview session was performed individually with each participant, using a demographic data form and an interview guide that structured the development of the interviews. This guide was designed in light of the existing literature and validated by two study collaborators^21^,^22^. The following guiding questions were included in the interview: How did you become a caregiver for your family member? What type of care have you provided so far? Do you feel you have enough knowledge to carry out caregiving tasks? Do you think you have developed the skills to provide home care so far? What do you need to be able to continue caring for your family member at home? How has the situation of being your family member’s caregiver affected your life? How long do you expect to be your family member’s caregiver? Is there anything else you’d like to share?.

Before data collection began, the interview guide was validated by experts and subjected to a preliminary pilot test with the purpose of assessing the relevance and coherence of the questions with the study's objectives. Both instruments were administered individually by the principal investigator in the hospital facility at a date and time previously agreed upon with the participant. A quiet, comfortable space was provided, with good ventilation, appropriate lighting, and the use of aromatherapy, ensuring the absence of interruptions.

To start the interview, participants were invited to engage in a guided, concentrative meditation. During this exercise, they were asked to visualize three key moments: a) the moment their family member suffered the stroke; b) the critical moments of the illness; and c) their first experiences as caregivers, both in the hospital and during the transition to home. The guided recollection aimed to prepare participants for deeper reflection on their caregiving experience, thereby facilitating interviews richer in detail and emotion.

After this reflective exercise, the first question was posed: How did you become your family member's caregiver? In-depth probes included: Could you explain how you did it? How did you feel at that moment? Could you tell me a bit more, or give me a specific example of what you did? From the first question onward, the rest of the questions were asked accordingly.

Before starting each interview, consent to audio-record the conversation was reaffirmed. To enhance data quality, notes were taken in the field diary and, at the end of the interview, the participants’ nonverbal cues were documented in this diary to provide additional information for the analysis and interpretation of the results. The interviews lasted between 30 and 60 minutes.

For data analysis, the Interpretative Phenomenological Analysis (IPA) proposed by Smith and Osborn was used^23^. The process was carried out in four stages:

1. Initial comments: Interviews were faithfully transcribed into Microsoft Word® to capture the essence of each conversation. Audio recordings were listened to multiple times, paying special attention to verbal expressions, pauses, and silences, while field notes were taken. Each transcript was carefully read and reread iteratively, to generate new insights—that is, new perceptions, understandings, or ideas emerging from the in-depth analysis of lived experiences. Initial comments were written in the margins of the transcript, and preliminary interpretations of relevant or significant aspects of each analyzed fragment were made.

2. Identification of emerging themes: The most important elements were grouped into themes and subthemes, and a thorough iterative examination of data was conducted.

3. Theme clustering: Patterns were sought that allowed the clustering of emerging themes, elevating the content to a higher level of abstraction in light of Edmund Husserl’s phenomenological framework^24^.

4. Summary of the study as a whole: A table of main themes and subthemes was developed, allowing the researchers to return to the original text for analysis as many times as needed.

When patients were admitted to the emergency department, the researcher approached the FC and would explain the research objective. For one week, rapport was built, and afterward the interviews were scheduled. Fieldwork concluded when two evaluators, through consensus, determined data saturation^21^.

To ensure the research’s trustworthiness, the criteria of credibility, auditability, and transferability were applied^25^. To ensure authenticity and veracity, data triangulation was used, which began with two authors of the study conducting inductive-deductive analysis, an analysis diagram, and an interview analysis report. These materials were then sent to a third researcher for further exhaustive analysis. The information was subsequently triangulated with the participant. A follow-up telephone meeting was scheduled, in which the results were presented and participants were asked to validate them. None of the participants requested any modification to the data.

Regarding the transferability criterion, all participants resided in Morelia, the capital city of Michoacán state, Mexico. The housing and infrastructure where the older adults lived consisted of well-developed urban homes. All caregivers were new to the caregiving role. A more detailed description of participant characteristics is presented in Table 1. The collected data is available for open access on Mendeley Data^26^.

**Ethical considerations**

The protocol was registered on the Electronic Registry System platform of the Health Research Coordination (SIRELCIS, by its acronym in Spanish) of the Mexican Social Security Institute (IMSS), under registration number F-2024-1602-030. It was subsequently approved by the Research Ethics Committee and the Research and Bioethics Committee, under registration number R-2024-1602-028. Participants were informed about the nature of their participation (risks, benefits, and authorization for audio recording) and provided written informed consent.

## Results

**Sociodemographic and occupational characteristics of family caregivers**

Six female caregivers participated. Two individuals who initially met the inclusion criteria and were interviewed were excluded from the study because the relatives they had cared for passed away, rendering verification of the collected data impossible.

All six participants reported being daughters of older adults with post-stroke sequelae, with a mean age of 53.67 years. Of these, 66.60% were married. Regarding educational level, 50.00% had secondary education, 33.30% had university studies, and 16.60% held technical qualifications.

Regarding occupation, 33.30% were homemakers, while 16.60% were retirees, accountants, employees, and self-employed workers, respectively, each representing 16.60%. Monthly income levels were generally low: most caregivers reported earning less than 400 USD, and some earned less than 150 USD. Dedication to caregiving varied: 50% of participants spent up to 24 hours a day on caregiving, implying continuous dedication, while the remaining caregivers dedicated between 5 and 8 hours per day. In addition, 50% of caregivers reported providing care for 6 to 7 days per week.

Housing and infrastructure where the older adults lived consisted of well-established urban homes. All caregivers were new to the caregiving role. A more detailed description of caregivers’characteristics is presented in Table 1. Regarding the characteristics of the stroke survivors, four had partial hemiplegia and two had total hemiplegia, with Barthel Index scores from 20 to 40 points, indicating severe dependence. Four survivors had dysphagia and aphasia and were fed via gastrostomy tube; only one had a tracheostomy.

The average duration in the caregiving role was 6.8 months. In terms of recreational and leisure activities, caregivers reported turning to activities such as watching movies, listening to music, going for walks, and crafting. A total of 83.30% of caregivers reported no use of harmful substances. Family and religious leaders were the main sources of support mentioned (Table 1).

**Table 1:** Sociodemographic characteristics of family caregivers of older adults with post-stroke sequelae

Family Caregiver Nickname	CF-Blue	CF-Black	CF-Orchid	CF-Three	CF-Puka	CF-PLight Pink
Gender	Woman	Woman	Woman	Woman	Woman	Woman
Age (years)	36	64	59	54	48	61
Education	University	Junior high school	University	Secretarial Technician	Hairdressing Technician	Junior high school
Marital status	Common-Law marriage	Married	Married	Married	Divorced	Married
Chronic-degenerative diseasess	Healthy	Hypertension-Rheumatoid arthritis	- Diabetes tipo 2. - Osteoporosis.	- Anxiety	Healthy	- Hypertension
Ocupation	Accountant	Homemaker	Retired	Employee	Temporary jobs	Homemaker
Socioeconomic status	$300 to 400 USD*	$300 to 400 USD*	$400 to 560 USD*	$300 to 400 USD*	(-) de $150 USD*	(-) de $150 USD*
Religion	Catholic	Catholic	Catholic	Catholic	Catholic	Catholic
Hours dedicated to caregiving	16 to 20 hrs	24 hrs	24 hrs	16 to 20 hrs	5 to 8 hrs	5 to 8 hrs
Days per week dedicated to caregiving	2 to 3 days	6 to 7 days	6 to 7 días	3 to 5 días	6 to 7 días	3 to 5 días
Time the caregiver has been caring for the older adult	6 months	6 months	6 months	9 months	8 months	6 months
Recreational or leisure activity	- Watch movies/ series - Crafting - Go for a walk -Listen to music	- Crafting - Go for a walk	- Crafting - Go for a walk	- Read books - Watch movies/ series	- Listen to music	- Go for a walk - Listen to music - Going out with friends to drink or dance
Consumption of harmful substances	No consumption	No consumption	No consumption	No consumption	- Alcohol - Tobacco	No consumption
Assistance resources	- Family - Religion - Financial	- Family	- Family - Friends	- Family - Psychological therapy	-Family	-Family

*USD = American Dollar; Hrs. = Hours; FC= Family Caregiver

**Figure 1:**
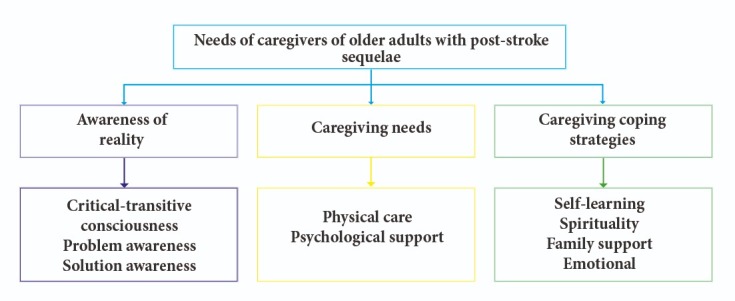
Core themes of meaning of caregivers’ needs

The methodological approach to phenomenology grounded in Edmund Husserl’s philosophical framework aims to generate a thorough, in-depth description of the personal beliefs and constructs reflected in participants’ discourse, based on their lived experience. In line with this approach, according to the assessment performed, three core themes of meaning that describe the caregivers’ needs emerged: Awareness of reality, care needs, and coping strategies for care. From these three main core themes, nine subthemes emerged, further describing their complexity (see Figure 1). 

*PICC: Peripheral inserted central catheter*

** 1. Awareness of reality**

**1.1 Critical-transitive consciousness**

Caregivers examine and become aware of their reality when they perceive the disability caused by the stroke in the older adult and the time required for their rehabilitation. This realization was striking, experienced with astonishment, concern, sadness, and uncertainty. Uncertainty emerges from the realization of home care needs, as they begin to feel incompetent (Table 2)

**Table 2:** Excerpts from the interviews alluding to awareness of reality

Subtheme	Excerpts
Critical-transitive consciousness	*“[…] When I entered the room, I saw my dad on the floor as if he were asleep, groaning. I tried to pick him up, but I couldn't. I grabbed his hand, and it was limp. I said to him, ‘Did you fall and break your hand?’ He just opened his eyes and moaned. I asked him, ‘Dad, does your head hurt?’ He tried to move his head, but he couldn't speak or move. I said, ‘Can't you speak or what?’ He just moa- ned, and then tears started to run down his face. Seeing him like that, I got really worried. When I saw that he couldn't speak, I thought he had a stroke. I began to cry out of worry.” (FC- Puka)* *“[…] When my dad woke up from the event, we realized that he could no longer communicate with us, so we tried to decode what he needed through his facial gestures. The question was, how would we know if he was in pain or if it got wor- se when we were at home?” (FC-Blue)* *“[…] in the emergency room, I began to see the disabilities my mother was ex- periencing, which made me realize that we couldn’t just let her die, because this wasn't going to be a one-day illness.” (FC- Three)* *“[…] they told us they were going to discharge him. So, I asked myself ‘What am I going to do at home to take care of him? How do I do the tasks they’ve told me to do?” (FC-Black)*
Problem awareness	*“[…] since I found out about the seriousness of my mother’s condition, I’ve felt scared and worried.” (FC- Three)* *[…] I’m aware of the situation. I lack the experience and knowledge to detect an emergency or the ability to take care of my husband, because something like this has never happened to us before.” (FC- Black)* *“[…] I have so many doubts. I wish the doctor would tell me if my husband is going to recover or explain to me what else will happen later. I need to unders- tand my husband’s prognosis for his recovery. His doctor only comes in the mor- ning to examine him and says everything is fine and that’s it. I see that he puts up a barrier so that no one can ask him anything. He doesn’t explain anything to me, and that discourages me from asking.” (FC-Black)* *“[…] the doctors haven’t confirmed how my mom will be after the stroke. I’m aware and understand they have a lot of work to do, and maybe that’s why they haven’t explained to us in detail what’s happening to her. We have many unanswered questions about her condition, especially because half of her body is numb, and we don’t know if she’ll be like this for life or if there’s any chance of recovery.” (FC- Three)* *“[…] Emotionally, I’m very sensitive seeing how my husband is ending up. I feel sad, I want to cry, seeing that he can’t move or speak makes me feel helpless and I’m extremely worried about how I’m going to handle this situation from now on.” (FC- Orchid)*
Solution awareness	*“[…] we need programs or places where people can share their experiences, express their emotions, and vent. It would be very helpful to have company and comfort in difficult times, especially when one feels alone, particularly in the early morning.” (FC-Blue)* *“[...] I think that, as a health system, they should support us more before dischar- ge and tell us how to care for our patient at home, because they never explained what we should do at home, and I think that’s why he got worse. I’m not, or we’re not, qualified to care for my husband at home.” (FC-Black)* *“[…] we need handbooks with drawings illustrating the steps of care routines so we can learn. It would be very helpful to have training once or twice a week at our home. I think that would be excellent because we already have a little more knowledge and skills.” (FC-Blue)* *“[…] we need a care handbook with illustrations so we can learn, because people like me, older adults, forget many things, in addition to training sessions once a week, until we feel prepared.” (FC- Orchid)*

**1.2 Problem awareness**

Caregivers are aware of the older adult's health problems and their own lack of knowledge regarding the progression and care they will need to provide at home, which generates feelings of powerlessness, uncertainty, sadness, fear, and worry. These emotions are intensified by the lack of communication between physicians and caregivers. Taken together, these feelings increase insecurity, a sense of inability, and frustration during the initial days of the transition from hospital to home care. 

**1.3 Solution awareness**

Awareness of reality also encompasses solution awareness. Although caregivers perceive themselves as incompetent to provide care, their experiences have led them to express the need for: 1) Mutual support programs in which they can share their experiences and emotions; 2) implementation of discharge plans that include caregiver training; 3) Illustrated handbooks for home care training; and 4) regularly schedule home visits for training and/or feedback to adequately address home care needs (Table 2). 

**2. Caregiving needs**

**2.1 Physical care needs**

After observing the hemiplegia resulting from the brain injury, caregivers experienced the need for training in physical rehabilitation, mobilization, hygiene, comfort, skin care, sleep quality, medication management, nutrition, and stimulation of gastrointestinal motility. Although these needs were accompanied by feelings of difficulty, helplessness, anguish, worry, frustration, and uncertainty, caregivers also expressed hope that by acquiring the skills needed to provide care, they could improve their relative’s quality of life and reduce the probability of hospital readmissions due to preventable complications (Table 3) 

**Table 3:** Excerpts from the interviews regarding caregiving needs

Subtheme	Excerpts
Physical care needs	*“[…] because of his body’s paralysis, I need to be taught how to do the rehabilita- tion exercises so he can get better soon. I feel that the sooner we start, the better it will be for him.” (FC- Black)* *“[…] the truth is, it’s hard for me to move her in bed to make her more comfor- table, or to prevent bedsores, I can’t do it alone, especially when I have to change her diaper. It’s important to take care of her genitals to prevent infections in the vagina, because she’s a woman, and well, it must be kept clean and dry.” (FC- Or- chid)* *“Bathing was the hardest part because I had to move him onto a plastic chair to pour water over him. He was weak for almost a month, so I could barely bathe him. I only used wet wipes.” (FC-Puka)* *“[…] apart from helping him move with someone’s assistance, bathing him daily, and keeping the bed clean, what else can we do to improve my dad’s well-being and comfort?” (FC- Blue)* *“[…] I try to feed him slowly and gently because he can’t swallow. It’s really hard to know what he can and can’t eat. (FC- Light pink)* *“[…] He lies motionless in bed, and we know he’s at risk of developing pressure sores because the mattress is hard plastic and also creates a lot of moisture. The question is, what else should we do besides moving him and putting cream to take care of his skin?” (FC- Three)* *“[…] now I see that I must learn how to take care of her. I’d like to be taught how to keep her awake, because she wants to sleep all the time, and it’s so frustrating and very complicated. (FC- Light rose)* *“[…] I know the basic medications he takes, but now they’re giving him several medications that I don’t know what they’re for. I need them to explain to me the purpose of each medication, how to give them, for how long, or if he’ll need to take them for life.” (FC- Orchid)* *“[…] I’d like to receive training on nutrition, that is, what to give him and how to feed him, that would make the issue of nutrition easier for me so that he reco- vers.” (FC- Orchid)* *“[…] at home, we realized he hadn’t gone to the bathroom for three days, so we called a doctor, and he explained to us that his digestive system wouldn’t function normally anymore, so we had to stimulate him to help him going to the ba- throom. At the hospital, they didn’t tell us anything about this.” (FC- Blue)* *“[…] We’d been home with him for two and a half weeks, but now I'm bringing him back because his stomach is swollen. They said he is constipated, so in the emergency room, they told us that we should do abdominal exercises. Still, no- body mentioned that before we left the hospital two weeks ago.” (FC-Black) * *“[…] after a week and a half at home, he got sick because he couldn’t go to the bathroom. They didn't tell me that he could become constipated.” (FC- Puka)*
Personal need for psychological support	*“Being at the hospital makes me anxious. I'd like to receive psychological therapy to help me cope with this situation because it has helped me in other situations.” (FC- Three)* *[…] this has been a very hard blow. I need a psychologist to listen to me. It’s not the same as having your family or friends listen to you because they get upset seeing you cry, so a professional knows and guides you to move forward. (FC- Orchid)*

**2.2 Personal need for psychological support**

Caregivers reported that the experience of their family member’s stroke affects their mental health. Consequently, they consider professional psychological support to be timely and necessary. Althou- gh they feel strengthened by their family and/or friends’ support networks, they say such support neither enough nor pertinent because expressing their emotions may negatively affect those who listen. In contrast, a psychologist possesses the knowledge, strategies, and experience to help them manage the stress and emotional burden associated with caring for their loved one (Table 3). 

**3. Caregiving coping strategies**

**3.1 Self-learning**

Caregivers develop coping strategies upon hospital discharge. In the absence of training, they un- dergo an intense self-learning process that involves observation, inquiry, and personal note-taking, which helps them sustain care at home. For caregivers, patient comfort is important; therefore, they opt for home-based care. Although they say they lack care training, they also demonstrate self-lear- ning through experience and willingness to learn. 

Self-learning through experience involves a trial-and-error process, and older adults’ body language has driven FC's creativity in interpreting their emotions and physical states through facial gestures, utilizing an emoji-based communication system. This symbolic communication system becomes a valuable resource for understanding their relatives’ well-being and adjusting care accordingly. This approach allows for a critical and reflective analysis of the environment and its influence on the person receiving care. In addition, caregivers’ children used technologies as a self-learning strategy for caregiving; instructional videos were a valuable tool for caregiving learning (Table 4). 

**Table 4:** Excerpts from the interviews illustrating caregiving coping strategies

Subtheme	Excerpts
Self-learning	*“[…] when they told us that he was going to be discharged, I started observing in the hospital how the nurses bathed him, changed his dia- per, and gave him medications. I brought a notebook and began writing down every day how they cared for my dad. Whenever I had a doubt, I asked the nurse, the doctor or the inhalation therapist why and how thigs were done, and I wrote everything down. That’s how we starting filling out the logbook with all the instructions for each care activity. […] The logbook we all created was a great help at home.” (FC- Blue)* *“[…] For the sick person, it’s better to be at home because they feel cal- mer. For us, it’s essential to know how to care for them because we don’t have the knowledge but we can learn little by little. We've figured things out from our experience at home. We didn't even know the name of what had happened to him.” (FC- Three)* *“[…] at home, I learned to take care of him little by little; I thought about ways to do things easier.” (FC-Puka)* *“[…] at home, we started relating his facial expressions to emotions. For example, if he’s happy, his eyes shine brightly; if he’s sad, he tends to look down; if he is angry, he frowns; and if he is desperate, he starts moving his hand and trying to take everything off. When we’re doing care activi- ties, we can tell from his gestures if we’re hurting him, if he’s in pain, or if he’s tired of being in that position. So, we also started using a list of emojis with all these meanings. So, if his face shows droopy eyes, then we have to ask the doctor for help because it means something hurts.” (FC- Blue) * *“[…] during these six months, we’ve done an unfailing caring job, but we’ve learned little by little on our own.” (FC- Blue)* *“[…] another thing we believe is that brightly colored places bring more lucidity. We put up blue curtains with fluorescent butterflies and placed him where a lot of light comes in; thus, his mood improves, as we see that he feels relieved when he sees those colors and that light. (FC- Blue) * *“[…] my children learned how to take care of their father through the Internet because no one was able to give us a brochure explaining how to do it. In fact, the hardest part was giving him his medication on the first day at home. So, my son watched a YouTube video on how to give medicine, and we found that crushing the tablets and dissolving them in water helped him swallow them faster and with less effort.” (FC- Black)*
Spirituality	*“[…] I believe that each person has their own destiny in life, and we must face circumstances and move forward. In my case, I accept the situation I've been given because there's no other alternative. I can only trust in God, who is the only one capable of giving us the strength to accept our circumstances. In my experience, God is the only one who grants resignation.” (FC-Puka)* *“[…] I pray to God to give me strength and fortitude until he leaves me. If God doesn’t take him, I have to stay with him. I won't abandon him. So, I'll wait until God decides to call him.” (FC-Orchid)* *“[…] I’ve always been a Catholic devoted to serving others because I like to help, and right now, that means helping my father. When my dad gets restless, we place a blessed Rosary on him, and we see that it calms him down because he handles the beads as if he were praying. I always ask God for everyone’s health, so my family can keep fighting alongside me to help my dad (FC- Blue)* *“[…] I want my mom to be well. I want her to live a long life and have the best quality of life possible. That’s my goal from now on. Taking care of her makes me feel good. I trust that everything will be okay.” (FC- Li- ght pink)* *“[…] I have confidence and a lot of faith that he’s going to recover, becau- se both he and we are putting in all our effort to move forward. I like to think I’m carrying a cross that doesn’t feel heavy, because I accept it with much love and heart, and I love my husband very much because he has been an excellent person to us.” (FC- Black)*
Family support	*“[…] it’s very hard to change his position every two hours, we can’t do it alone. My dad’s weight is too much, so we realized we need at least two people to do everything. That way, it’s less stressful for us.” (FC- Blue) * *“[…] we’re nine siblings, and we’ve always been very close, so we all lear- ned to take care of my dad.” (FC- Blue)* *“[…] my children and I decided to help each other so we could care for him at home and make it less burdensome, and thus be able to move forward with him. But the one who takes the most responsibility is me, his wife. (FC-Black)* *“[…] we all took care of my husband with a lot of love so it wouldn’t feel so awful and burdensome.” (FC-Black)*

**3.2 Spirituality**

 In caregivers’ accounts of their experiences, spirituality and religiosity are seen as a coping strategy. They accept their caregiving role as a divine purpose, which they assume with a sense of surrender and, at the same time, acceptance and commitment. The former because they feel they have no op- tion but trust in a higher power, and the latter because they believe that, as it is a divine purpose, they will receive strength. This spiritual perspective provides a sense of peace and purpose amid adversity.

 Caregivers practice prayer as a means of spiritual connection with a higher power, seeking strength to continue caregiving with the conviction that their prayers will be heard. When the older adult participates in these religious practices, caregivers perceive a shared sense of calm and serenity. The act of caregiving itself generates well-being and hope, rooted in love and reciprocity. Through their caregiving role, they find purpose and meaning in life, which in turn contributes to their well-being and hope (Table 4).

**3.3 Family support**

 Shared family responsibility emerges as a crucial resource for addressing caregiving needs, yielding positive effects by reducing the mental and physical burden on the primary FC. Caregivers express that their families assumed the caregiving role by choice, within an environment of unity and family affection, which helps prevent caregiver burden. Sustainable family caregiving is essential to facing the challenges of long-term caregiving and creating a strong and emotionally meaningful support network (Table 4). 

**3.4 Emotional coping strategies**

The testimonies reflect the emotional impact experienced by FCs in caring for older adults with post- stroke sequelae. For many FCs, caregiving has been an exhausting task, often accompanied by feelings of helplessness, sadness, psychological pain, and burden. Caregiving needs have had a negative emotional impact on caregivers, and the lack of care training and preparation further exacerbates fear, stress, a sense of difficulty and helplessness, and anxiety. Despite the physical and emotional exhaustion associated with their caregiving role, family unity, reciprocity, and love are present as compensatory strategies that help caregivers cope with the challenges of home-based care (Table 5).

**Table 5:** Excerpts from the interviews allusive to the impact of needs

Subtheme	Excerpts
Emotional	*“[...] I see my dad as much more fragile now. He’s in a vegetative state. The doc- tors tell me there’s nothing that can be done, and it’s like going back to the past. Honestly, it’s so exhausting. I feel tired, not just physically, but emotionally, from thinking about everything I’ve gone through with him since his first stroke. I feel like I can’t do it anymore.” (FC- Puka)* *“[...] emotionally, it’s so sad to see my dad suffer every single day. It hurts my soul and breaks me into pieces, because I can’t assimilate it or understand it, and I can’t help him any more than I already do.” (FC- Blue)* *“[…] We feel so afraid because we really don't know what to do.” (FC- Blue) * *“[…] Taking care for my husband at home is very difficult because I don’t have the knowledge, I don't know how to do things properly, and all this caregiving situation causes me a lot of stress.” (FC-Black)* *“[…] my mom has had two strokes, but this time it was very serious and it affec- ted her badly because half of her body lost all strength. Now I feel like it’s going to be hard for me to provide care. I feel like it’s going to be very difficult, and I actually feel quite stressed.” (FC- Light pink)* *“[…] we’ve learned and faced the situation with love.” (FC- Blue).* *“[…] The truth is, I feel tired, but the love I feel is greater than my tiredness.” (FC- Black)* *“[…] now that my mom needs me, I’m willing to give back a little of the love she gave us. Thank God I’m not the only caregiver; there are four of us, all women, and we all help each other.” (FC- Three)*

## Discussion

In the different forms of consciousness evoked by FCs, the sense of their experience, based on intentionality, is implicit^19^,^27^. The FCs reported awareness of different realities, such as the severity of the health condition of the older adult with stroke, their perceived lack of knowledge and skills to provide care at home, and the need for training from healthcare professionals to improve their loved one’s health conditions.

Several authors, such as Capelo et al,^28^ have noted that FCs have limited knowledge for providing comprehensive care, making it imperative for health professionals to develop inclusive and humanized educational strategies aimed at strengthening the caregiver's role. In this context, it is essential to avoid attitudes of professional arrogance^29^, which, as Ramos et al^30^, warn, hinder the construction of collaborative relations by perpetuating traditional models focused exclusively on technical aspects, leaving aside the ethical and affective dimensions of care. Similarly, Pontón et al.^31^ show that manifestations of hierarchy and lack of empathy represent one of the main barriers to effective communication between healthcare professionals and caregivers, negatively affecting the therapeutic relationship. Promoting a humanized education, therefore, involves recognizing the caregiver as an active subject, someone with the potential to learn, make decisions, and consciously engage in the caregiving process. This recognition strengthens caregivers’ self-management, as well as their solution-focused awareness. From Husserl’s phenomenological perspective, this intentional awareness of the“need for training”can even emerge as an anticipatory lived experience or a projective representation of the caregiver’s desire to provide better care^32^.

Within solution awareness, participants in this study described various self-learning strategies to expand their caregiving knowledge and skills. This situation is present in other studies^15^,^33^areporting that caregivers turn to the internet to learn about stroke, its symptoms, risk factors, treatment, rehabilitation, and care. This pattern reinforces the trend among caregivers toward complementing observational training with digital resources.

Regarding identified needs, these emerged from the perceived realities regarding the lack of knowledge about the various aspects of home care required by older adults after stroke. These needs ranged from basic aspects, such as hygiene and nutrition, to pharmacological and rehabilitation aspects, without excluding aspects such as spirituality, resilience, and coping with a chronic and debilitating illness. In this process, FCs support the older adult while also managing their own feelings, which implicitly results in caregiver burden and, consequently, a need to be heard and cared for by health professionals.

Stressful reactions resulting from lack of knowledge about the specific care older adults require for certain illnesses have been previously described by health professionals^34^. However, this study presents these needs from the FCs’ lived experiences, which may contribute to the joint resolution of two interconnected health issues: the health of older adults with stroke and the prevention of caregiver burden. There is evidence that it is necessary to consider the target population’s needs to understand the phenomenon better and help health professionals implement appropriate interventions, which, in turn, fosters better adoption of these interventions by users. In this sense, other authors highlight the importance of educational programs for people with chronic illnesses that also support the caregiver role, strengthening self-care^35^.

Understanding the FCs’ needs may enable healthcare personnel to broaden their care perspective and improve communication processes, thereby removing barriers that arise during the preparation for hospital-to-home transitional care^4^. Such understanding may also foster the development of introspective and critical skills that allow them to connect with the patient's subjectivity, integrating ethical and philosophical aspects into their work and promoting a humanized, empathetic approach^27^,^32^.

Awareness of the diverse realities surrounding their family member’s health motivates FCs to develop strategies to cope with or improve these realities, drawing on their ingenuity to adapt to new ways of living, communicating, and relating with the person they care for. This finding invites healthcare professionals to value the role of FCs in maintaining health and to recognize them as a key component of secondary prevention^36^.

Confronting this new way of life, with a greater burden of negative feelings, responsibilities, and daily activities, prompts FCs to seek out strengths, such as spirituality and family support, as coping strategies. Through spirituality, FCs strengthen faith and trust in the divine, helping them process emotions, reduce tension, and find meaning in their role^37^,^38^.

This strategy enables FCs to provide not only physical and psychological support, but also to accompany patients in their search for meaning. Through phenomenological reflection, caregivers can explore their own transcendental dimension, finding purpose and connection with something greater, which helps them face the difficulties of their role in a positive way^39^,^40^.

Likewise, it has been noted that, in spirituality, caregivers find strength and faith to continue, which serves as emotional support during the adaptation process, helping them understand their role and affirm its dignity. For Husserl, spiritual support is fundamental in caregiving, as it respects the caregiver's transcendence and meaning in life^38^. By integrating spiritual well-being, spiritual help offers comprehensive support that connects individuals to a higher purpose^32^.

Regarding family support, collaborative caregiving arises from the need to distribute roles within the family network, which facilitates the most demanding tasks —such as mobility, transfers, and hygiene— reducing stress and complexity of caregiving. These results are consistent with those of Denham et al.^9^ and Wang et al.^41^, who found that caregivers who request support from their family networks do so to reduce caregiving burden and to provide quality care^42^. Supplementing the caregiver's role reduces stress and strengthens the caregiver's well-being^42^,^43^.

From a phenomenological perspective, collaborative work is based on “intersubjectivity”, where the unique experiences of each caregiver are valued. According to Husserl, this approach facilitates shared knowledge that enriches caregiving practice and alleviates the emotional burden by distributing responsibilities and sharing lived experiences^27^,^32^.

## Conclusions

The needs arising from the experience of FCs of older adults with post-stroke sequelae include the need for training to provide basic and complex care at home in response to, and in light of, the older adult’s physical sequelae, as well as the need for psychological support to manage the negative effects of home care effectively.

Caregivers initially experience feelings of inadequacy, which are exacerbated by a lack of communication with physicians and the absence of appropriate training, leading to heightened insecurity and frustration.

Despite these challenges, FCs identify essential strategies and needs to address their role. Caregivers reported the need for coping strategies such as spirituality, which emerges as a refuge grounded in faith and spiritual beliefs that helps them make sense of the situation, find inner strength, and cope with anxiety and hopelessness. Furthermore, family support plays a dual role: it provides practical assistance and preparation for home care, while also fostering emotional and logistical involvement of other family members
